# Microencapsulation of β-Glucosidase in Alginate Beads for Post-Rumen Release in Ruminant Gut

**DOI:** 10.3390/bioengineering12121341

**Published:** 2025-12-09

**Authors:** Nada Almassri, Francisco J. Trujillo, Athol V. Klieve, Robert Bell, Danyang Ying, Netsanet Shiferaw Terefe

**Affiliations:** 1 Commonwealth Scientific and Industrial Research Organisation (CSIRO), 671 Sneydes Road, Melbourne, VIC 3030, Australia; nada.almassri@csiro.au (N.A.);; 2 School of Chemical Engineering, The University of New South Wales, Sydney, NSW 2052, Australia; 3 ProAgni Pty Ltd., Albury, NSW 2641, Australia

**Keywords:** biopolymers, enzyme stabilization, gastrointestinal delivery, microencapsulation optimization, rumen bypass microcapsules

## Abstract

This study aimed to develop a microencapsulation formulation for efficient encapsulation of β-glucosidase to improve its stability in a rumen-like environment and sustain activity post-rumen in the ruminant gut. Various alginate-based formulations were evaluated to achieve high encapsulation efficiency (EE) and stability. These included control alginate beads (AB), microcapsules with chitosan (MCS), alginate–sucrose beads (AOS), alginate–sucrose–maltodextrin beads (AOMS), and alginate pectin beads (APB). The microcapsules were made using Buchi encapsulator B-390 with calcium chloride as the gelling solution. Alginate proved to be a suitable polymer for β-glucosidase encapsulation and <1 mm diameter microbeads were obtained across all formulations. Alginate alone (AB: 1% alginate, 0.2 U/mL β-glucosidase) showed low EE (3% ± 1.0) due to leakage and syneresis. Modifying the gelling solution with 0.1% chitosan (MCS) increased EE to 49 ± 2.64% by reducing alginate porosity. Further improvements were achieved by adding stabilizers to the alginate solution (AB), in addition to using the modified gelling solution (MCS): Adding sucrose (AOS) at 4% increased EE to 95.5 ± 2.08%, while adding sucrose (4%) and maltodextrin (2%) (AOMS) achieved 100 ± 2.16%. On the other hand, adding pectin (4%) (APB) to the alginate solution resulted in a lower EE of 40.5% ± 2.55, likely due to interference with alginate crosslinking. In vitro rumen fermentation showed a dry matter degradation of 42–54%, underscoring the need for more robust microcapsules. Encapsulation strategies, such as incorporation of additional protective layers, are essential to enhance bead stability, minimize degradation, and improve enzyme retention, to ensure efficient delivery and sustained enzymatic activity in the hindgut.

## 1. Introduction

The inclusion of fibre-degrading enzymes in the ruminant feed can potentially improve residual fibre digestibility and feed utilisation rate [1,2]. However, the varying pH levels throughout the ruminant digestive tract from alkaline in the mouth, to mildly acidic or neutral in the reticulorumen and omasum, to highly acidic in the abomasum, and then back to alkaline in the small intestine pose a significant challenge to maintaining the enzymes in their active form during delivery [3]. In addition, the rumen’s complex microbiome and the enzymes secreted by these microbes can cause the denaturation and inactivation of the fibre-degrading enzymes [3,4]. Microencapsulation can help deliver sensitive materials such as nutrients and enzymes to various locations in the gastrointestinal tract (GIT) of ruminants [3] preserving their activity, and enabling controlled release [5].

In recent years, there has been an increasing interest in utilizing polymer-based capsules derived from natural sources. Alginate capsules offer selective retention and controlled release properties, making them highly suitable carriers for various biotechnological applications [6]. Alginate beads are frequently used in the encapsulation process as an entrapment matrix for enzymes and cells as they have the following advantages: (i) they are nontoxic, (ii) are low cost, (iii) resistant to microbial attack, (iv) easy to formulate, and (v) encapsulation is carried out under mild conditions [7,8]. In addition, acid sensitive enzymes entrapped in the beads are protected from stomach acidity and gastric juice [8]. On the other hand, alginate beads have significant limitations, including wide pore size, considerable enzyme leakage, low mechanical strength, low encapsulation efficiency and the gel formed is susceptible to high pH levels which can affect both the release and protection of the encapsulated material [6,9]. In addition, chelating agents like lactates, citrates, and phosphates can destabilize this network [10]. Nevertheless, these shortcomings can be potentially overcome by coating alginate beads with additional wall materials such as chitosan, which could decrease the porosity of the alginate matrix, enhance its structural integrity and prevent enzyme leakage [9,11,12].

Lipid and protein-based microencapsulation systems can also be potentially used for the delivery of actives to the ruminant gut. For example, encapsulation of methionine using carnauba wax matrix provided excellent protection in the rumen because of its hydrophobic nature [13]. Among lipid-based strategies, liposomes have gained attention for their ability to encapsulate hydrophilic and hydrophobic compounds. For example, rumen-protected microencapsulated fatty acids derived from linseed oil which was evaluated for its stability in the rumen and its impact on milk composition in dairy cows. The encapsulation technique successfully protected the fatty acids from microbial degradation in the rumen, allowing for their gradual release and intestinal absorption. This was reflected in a measurable increase in omega-3 fatty acid content in the milk [14]. However, such matrices are not suitable for heat-sensitive compounds such as enzymes since they could be damaged/denatured during the encapsulation process, which is conducted at high temperature. The resulting coatings may also hinder the complete release of the encapsulated enzyme in the lower gut. Protein-based systems have also been evaluated for the delivery of actives into the ruminant gut. For instance, Yoshimaru, et al. [15] evaluated zein based formulation for encapsulating L-Lysine. The encapsulation provided ruminal protection and passage through the rumen with minimal degradation. However, about 90% of the encapsulated L-lysine was released in simulated abomasal fluid likely due to the combined effects of acidic pH and pepsin activity in the abomasal environment [16,17]. Additionally, a recent study by De Jesús, et al. [18] investigated the use of water-in-oil-in-water (W/O/W) double emulsions for encapsulating amino acids. The findings suggested that the release of the amino acids and dipeptides was primarily influenced by their solubility in the oil phase, pointing to partial protection in the rumen. However, the study also identified physical instability as a key limitation, as phase separation and leakage were observed over time. Agarose-based immobilization has shown promising enzyme retention and lignin digestibility improvements, particularly with laccase. However, this approach is not yet practical for large-scale feed applications due to cost and formulation complexity [19].

Chitosan, a β-1,4 linked linear polymer of 2-acetamide-2-deoxy-β-D-glucose, is a natural polymer that is non-toxic, biodegradable, and biocompatible. It has a broad range of applications, including in biomedicine, membranes, drug delivery systems, hydrogels, water treatment, and food packaging [9,12]. The cross-linking of alginate and chitosan in hydrogels creates materials with enhanced stability compared to those obtained with a single polymer, making them suitable for medical and pharmaceutical applications. In recent years, alginate–chitosan polyelectrolyte complexes have attracted considerable attention in controlled drug delivery. These complexes are formed through ionic interactions between the carboxyl groups of alginate and the amino groups of chitosan [12,20].

In this study, we assessed the feasibility of using alginate-based beads for the microencapsulation of enzymes aimed at protected delivery to the ruminant hindgut, with beta-glucosidase selected as a model enzyme due to its key role in the hydrolysis of cellulose within fibre-rich ruminant diets. We hypothesized that the incorporation of chitosan into the bead formulation would address some of the limitations of traditional alginate-based systems. Previous studies have shown that chitosan can improve encapsulation efficiency and protect enzymes from environmental stressors [9,11], while the addition of carbohydrates such as sucrose and maltodextrin would enhance enzyme stability during the drying process [21]. Sucrose acts as a glass-forming disaccharide that stabilises protein structures by replacing water molecules and preserving the native conformation of the enzyme, thereby reducing denaturation and aggregation during encapsulation and subsequent drying steps [22,23]. Maltodextrin, on the other hand, functions as a film-forming polysaccharide providing structural bulk and reducing internal stresses within the bead matrix [24,25]. Its ability to lower water activity and increase matrix viscosity further contributes to improved encapsulant retention and reduced leakage during processing [26,27]. Pectin was incorporated to reinforce the alginate network and reduce bead porosity, thereby limiting diffusion and improving the retention of encapsulated compounds [28]. Therefore, we evaluated the impact of these components on the stability of beta-glucosidase, which is involved in the hydrolysis of cellulosic fibres in the ruminant hindgut. The formulations were characterised using a combination of encapsulation efficiency (EE), dry matter disappearance (DMD), swelling ratio measurements, enzyme activity recovery (ER) following simulated ruminal incubation, and structural evaluation through scanning electron microscopy (SEM). To date, no studies have explored the use of alginate beads for enzyme delivery to the ruminant hindgut for fibre digestion. This study addresses this gap by investigating the potential of alginate-based beads for enzyme delivery specifically to the ruminant hindgut.

## 2. Materials and Methods

### 2.1. Materials

β-Glucosidase (*Agrobacterium* sp.) (EC 3.2.1.21) was purchased from Neogen Australasia Pty Limited (Queensland, Australia), *p*-nitrophenyl-β-D-glucopyranoside (PNPG, ≥99% purity; CAS 2492-87-7), Chitosan (CAS 9012-76-4), CaCl_2_ (≥99.0% purity; CAS 10035-04-8), Citric acid trisodium salt (≥98.0% purity; CAS 68-04-2), *p*-nitrophenol (CAS 100-02-7), 4-(2-hydroxyethyl)-1-piperazineethanesulfonic acid (HEPES, ≥99.5% purity; CAS 7365-45-9) and Na_2_CO_3_ (≥99.5% purity; CAS 497-19-8) were purchased from Sigma (Sydney, Australia). Shellac (Swanlac ASL 10) was supplied by A.F. Suter & Company Ltd., (Witham Essex, UK). Sodium alginate (E401, C_6_H_9_NaO_7_, Chile origin) with a molecular weight of 216.121 g/mol and maltodextrin (DE18) were purchased from The Melbourne Food Depot (Melbourne, Australia), and sucrose (CAS 57-50-1) from Thermo Fisher Scientific (Scoresby, Australia). Tween 20 was purchased from Acros Organics (CAS 9005645, Geel, Belgium), and sodium alginate (Protanal SF120RB, C_6_H_9_O_7_) from FMC Biopolymer (Sydney, Australia). The McDougall buffer was prepared by mixing the following materials [29], all purchased from Sigma (Sydney, Australia): (9.8 g/L sodium bicarbonate (NaHCO_3_; CAS 144-55-8), 4.65 g/L disodium hydrogen phosphate dihydrate (Na_2_HPO_4_.2H_2_O; CAS 10028-24-7), 0.57 g/L potassium chloride (KCl; CAS 7447-40-7), 0.47 g/L sodium chloride (NaCl; CAS 7647-14-5), 0.12 g/L magnesium sulfate heptahydrate (MgSO_4_.7H_2_O; CAS 10034-99-8), and 0.04 g/L calcium chloride (CaCl_2_; CAS 10035-04-8).

### 2.2. Enzyme Activity Assay

The enzyme activity was assayed following the method of Terefe, et al. [30] with some modifications. The reaction mixture consisted of 200 µL of 0.2 unit/mL of β-glucosidase in HEPES buffer (pH 6.8), 500 µL of HEPES buffer (pH 6.8), and 200 µL of 0.35 mM *p*-nitrophenyl-β-D-glucopyranoside (PNPG) solution in HEPES buffer (pH 6.8). The reaction mixture was incubated for 24 h at 40 °C, followed by boiling for 4 min to inactivate the enzyme and cooling in ice-water for 10 s. Subsequently, 100 μL of 0.18 M Na_2_CO_3_ was added to the reaction mixture to stop the reaction and develop the colour, and the absorbance at 405 nm was measured using a UV-Vis spectrophotometer (UV-1700, Shimadzu, Kyoto, Japan). A standard curve was used to determine the concentration of the reaction product, *p*-nitrophenol (PNP), from the absorbance data and the activity of the enzyme was expressed as µmole of *p*-nitrophenol produced per min under the reaction condition. Using a series of PNP standards (0.6 to 85 µM) in a 1 mL reaction mixture containing the same concentration of Na_2_CO_3_ in the final assay mixture, the standard curve was generated as shown in Figure S1 in the Supplementary Material.

### 2.3. Preparation of Encapsulation Solution

A 1% (*w*/*v*) sodium alginate solution (E401) was prepared by mixing alginate powder with distilled water while stirring at 500 rpm overnight at 4 °C using a magnetic stirrer. The solution was then refrigerated for 24 h to enable hydration and to remove any air bubbles that were introduced during agitation [31]. All polymer solutions were prepared 24 h prior to their use. Fifty microliters of the enzyme were diluted in 100 mL of HEPES buffer solution (50 mM, pH 6.8). The diluted β-glucosidase solution was then mixed with the alginate solution to achieve a final enzyme concentration of 0.2 U/mL under constant stirring. The alginate-β-glucosidase mixture was transferred into the feeding bottle, and the capsules were formed as described in Section 2.4.

### 2.4. Production of Alginate Beads Using B-390 Encapsulator

**Figure 1:**
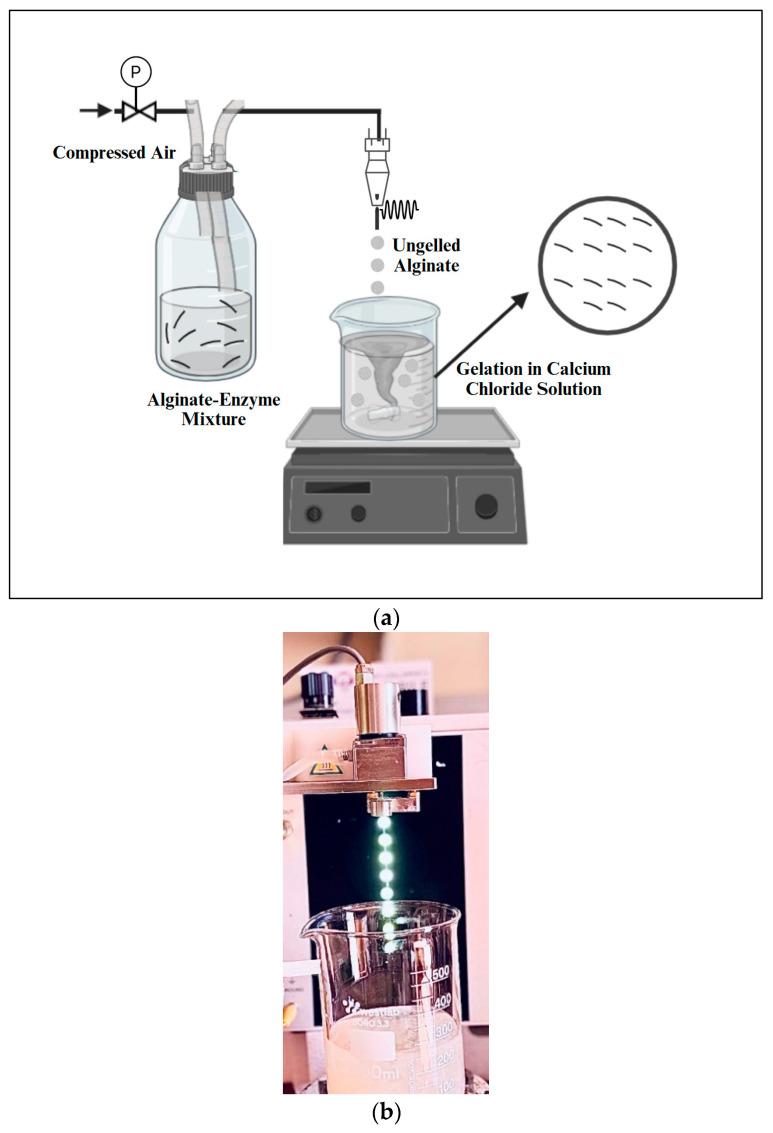
Alginate Microbead Production Process (**a**) Schematic Representation of Alginate–Enzyme Bead Formation Process; (**b**) Buchi Encapsulator B-390 (Buchi, Flawil, Switzerland) Setup for Microbead Formation. Alginate microbeads were produced using a Buchi Encapsulator B-390 with a 750 µm nozzle, formed under a vibrational frequency of 40 Hz, and hardened in 0.1 M calcium chloride for 30 min.

Alginate microbeads were produced using Buchi encapsulator B-390 (Buchi, Flawil, Switzerland) (Figure 1), which was equipped with a 750 µm nozzle as described by Siang, Wai, Nyam and Pui [10] with some modifications. The alginate–enzyme mixture (1% (*w*/*v*) sodium alginate E401, 0.2 U/mL β-glucosidase) in the feeding bottle was pumped into the encapsulator and extruded through a 750 µm nozzle to form small droplets. The external pressurized air supply was activated to pressurize the feeding bottle, with the pressure set to 530 mbar. A vibrational frequency of 40 Hz was applied during the pumping of the alginate–enzyme mixture to create microcapsules by atomizing the jet into uniformly sized droplets. The droplets were dropped into the crosslinking 0.1 M calcium chloride solution and allowed to harden for 30 min at 4 °C. After this gelation period, the formed capsules were washed with 1 L MilliQ water using a filter bag to remove any excess calcium chloride solution from their surfaces before collection [9,32]. This was followed by drying the beads at room temperature for 24 h and storage at 4 °C until further analysis [9].

### 2.5. Determination of Encapsulation Efficiency (EE%)

The encapsulated enzyme was released by dissolving weighted beads in a trisodium citrate solution (0.2 M, pH 8.2). The resulting mixture was subsequently analyzed to determine the enzyme activity recovered from the beads [9,11] using the assay described earlier. The encapsulation efficiency was determined by calculating the activity of the encapsulated β-glucosidase, enzyme activity recovered from the beads, and expressing it as a percentage of the initial β-glucosidase activity in the alginate–enzyme mixture as described in Equation (1) [9]. Experiments were performed in triplicates, and the results were the average of three independent data points.

(1) $$\mathrm{EE}\;(\%)=\frac{\mathrm{Enzyme}\;\mathrm{activity}\;\mathrm{recovered}\;\mathrm{from}\;\mathrm{the}\;\mathrm{beads}}{\mathrm{Initial}\;\mathrm{Enzyme}\;\mathrm{Activity}\;}\times 100$$

where the initial enzyme activity is that in the alginate–enzyme and alginate–copolymer–enzyme solution.

### 2.6. Incorporation of Polymers and Carbohydrate Stabilisers

The porous nature of alginate capsules highlighted the need for optimization of the encapsulation process and encapsulant formulations. Thus, various formulations were explored in order to reduce the porosity of the alginate beads and improve the encapsulation efficiency of the enzyme. The formulations are summarized in Table 1. In all cases, the concentration of β-glucosidase was maintained at 0.2 U/mL. The cross-linking solution was also modified by adding chitosan (0.1% *w*/*v*) and Tween 20 into the crosslinking solution (CaCl_2_) in accordance with the method of Anjani [11] and Thu and Krasaekoopt [9] with some modifications. Accordingly, 5 mL of acetic acid was added to 1 g of low-molecular-weight chitosan (50,000–190,000 Da), and the mixture was diluted with deionized water to a total volume of 700 mL. Subsequently, CaCl_2_ solution (0.1 M) and Tween 20 (0.1%) were added to the chitosan solution. The final volume was then made up to 1000 mL with deionized water, yielding a chitosan solution at a concentration of 0.1% (*w*/*v*) in 0.1 M CaCl_2_ with 0.1% Tween 20 (Formulation (2), Table 1). Tween 20 was used to reduce the surface tension of the gelling water to facilitate the formation of spherical beads [9].

The bead formulations were modified by incorporating 4% sucrose (Formulation (3)), incorporation of 4% sucrose and 2% maltodextrin (Formulation (4)) and incorporation of 4% pectin (Formulation (5)), with the concentration representing the final values in the formulations. The various carbohydrate stabilisers were evaluated as they have the ability to enhance encapsulation efficiency by providing a protective environment that reduces protein denaturation during the encapsulation process [9,33]. High-viscosity alginate (E401) was used in all the four formulations at 1% (*w*/*v*) with a measured viscosity of 151 cps.

### 2.7. In Vitro Rumen Fermentation to Assess the Stability of the Encapsulated Enzyme

#### 2.7.1. Preparation of Microencapsulated Enzyme Samples

Based on the results of the exploratory experiments in Section 2.6, the bead formulation incorporating maltodextrin and sucrose was selected for the in vitro rumen fermentation study. In this case, two types of alginate polymers were evaluated; low and high-viscosity alginate so as to understand the effects of alginate viscosity on enzyme protection. A 2% (*w*/*v*) solution of low-viscosity alginate (SF120RB) and a 1% (*w*/*v*) solution of high-viscosity alginate (E401) were prepared by mixing alginate powder with distilled water under agitation at 500 rpm overnight using a magnetic stirrer. To each solution, maltodextrin (DE-18), sucrose, and an enzyme solution were added to obtain total solids of 2% and 4%, respectively, for high- (HVAB) and low-viscosity beads (LVAB), and 0.2 U/mL enzyme concentration (Table 2). For crosslinking, a solution containing 0.1 M CaCl_2_, 0.1% chitosan, and 0.1% Tween 20 was used (Table 2). The beads were prepared as described in Section 2.4. Control beads without enzyme were also produced in the same way to serve as blanks for measuring the enzyme activity referred to as LVABc and HVABc.

#### 2.7.2. Collection and Pre-Incubation of Rumen Fluid

Five hundred millilitres of rumen fluid were collected per run from late-lactation cannulated Holstein Friesian dairy cows, aged 4, 5, and 7 years, at Agriculture Victoria (Ellinbank, Australia) in the morning before feeding and transported to the laboratory as described by Tunkala, et al. [34] and Tunkala, et al. [35]. Cows were grazing a solvent-extracted canola meal, and wheat and barley grain mix (6.1 kg dry matter (DM) per day per cow) that was supplied in the milking parlor. The rumen fluid was transported to the laboratory in a pre-warmed 1 L glass bottle placed in an incubator (Galaxy 14 S, New Brunswick Scientific, Eppendorf, Edison, NJ, USA) set to 39 °C. The bottles were sealed with a one-way gas valve to permit the release of generated gas. Upon arrival at the laboratory, the rumen fluid was filtered through two layers of cheesecloth to separate the liquid from the residual solids. The liquid fraction was subsequently transferred to a pre-warmed 1 L glass bottle, flushed with CO_2_ for one minute at a pressure of 100 kPa and a flow rate of 20 L/min, and utilized as fresh rumen fluid (RF) for incubation on the day of collection. This experiment was conducted on three independent trials on different days, and the data presented in this study are from the final trial.

#### 2.7.3. Fermentation and Experimental Design

Using the method of Tunkala, DiGiacomo, Alvarez Hess, Dunshea and Leury [35] with some modifications, the pre-incubated rumen fluid was mixed with McDougall’s buffer ‘synthetic saliva’ (pH 6.8 ± 0.1) to obtain a buffered rumen fluid with a 1:3 rumen fluid to buffer ratio. The feed sample, Oaten Chaff, (Petstock, Ballarat, Australia) were ground using a domestic grinder (Breville, Sydney, Australia) and sieved by a 1 mm size sieve. A filter bag with a pore size of 25 µm (F57 ANKOM bag, ANKOM Technology, Macedon, NY, USA) was utilized to measure the dry matter disappearance (DMD) of the incubated sample [34,36]. For Rumen fluid analysis on DMD and encapsulation efficiency, heat-sealed F57 filter bags were prepared with each bag containing one gram of either LVAB or HVAB. Control filter bags containing one gram of LVABc or HVABc were similarly prepared. These filter bags were then incubated in triplicate in 250 mL glass bottles with 1 g of feed sample (Oaten Chaff) and 100 mL buffered rumen fluid for 24 h in water bath maintained at 39 °C and 50 rpm (Julabo, SW23, John Morris Scientific, Sydney, Australia) [34,36]. For buffer analysis on DMD and encapsulation efficiency, one gram of LVAB and one gram of LVABc were placed in heat-sealed F57 filter bags. These bags were incubated in triplicate in 250 mL glass bottles with 1 g of feed sample (Oaten Chaff) and 100 mL of MacDougall Buffer for 24 h in a 39 °C/50 rpm water bath. For background analysis, one gram of feed sample was weighed in 250 mL glass bottles, with 100 mL buffered rumen fluid dispensed to the bottles using a liquid dispenser and incubated in triplicate for 24 h in a 39 °C/50 rpm water bath. All glass bottles were purged with CO_2_ prior to incubation, and the tube was subsequently sealed with a rubber bung [34,37]. After 24 h of incubation, the filter bags were gently rinsed with distilled water using a plastic water diffuser (tattoo squeeze). The swollen beads were weighed immediately after the removal of the adhering liquid. The swelling rate of the beads was calculated using Equation (2) [36].

Swelling rate (%) = ((Wt − Wo)/Wo) × 100%(2)

where Wt is the weight of swollen beads at time t, and Wo is the initial weight of the beads.

The residue was dried in a fume hood at room temperature for 24 h. The samples were weighed to determine the dry matter disappearance (DMD) as a measure of degree of degradation, which was calculated from the initial weight using Equation (3) [34,38].

DMD (%) = ((W1 − W2)/W1) × 100(3)

where W1 is the initial dry weight (g) of the beads before ruminal incubation, and W2 is the weight (g) of the final air-dried beads after ruminal incubation.

The pH value of the buffered rumen fluid was measured pre- and post-fermentation using a pH meter (WP-81, TPS). Enzyme recovery (ER) was evaluated as illustrated in Equation (4). To evaluate enzyme recovery pre- and post-ruminal incubation, weighted beads were dissolved in a trisodium citrate solution (0.2 M, pH 8.2). This dissolved mixture was analyzed for enzyme activity using enzyme assay as previously described [9,11].

(4) $$\mathrm{ER}\;(\%)=100-(\frac{E0-E1}{E0\;}\times 100)$$

where E0 is the enzyme activity before ruminal incubation, and E1 is the enzyme activity after ruminal incubation.

#### 2.7.4. Morphological Characterization of Hydrogel Microbeads

To examine the structure of the microcapsules, the samples were mounted on aluminium stubs using double-sided conductive carbon tabs. These samples were then coated with iridium using a Cressington 208HRD sputter coater (Cressington Scientific Instruments Ltd., Watford, UK), achieving a coating thickness of approximately 6 nm (60 mA for 60 s). Conductive coating is essential to prevent charge accumulation in an electron microscope, ensuring clear images, particularly for insulating materials [39,40]. The samples were imaged using a Zeiss Sigma VP300 FESEM (Field Emission Scanning Electron Microscope, ZEISS, Oberkochen, Germany). Images were captured in secondary electron (SE) mode, which highlights topographical features [39]. An accelerating voltage of 5 kV was utilized for imaging. All samples were imaged under the same magnification (30×).

#### 2.7.5. Stability of Encapsulated Enzyme in the Beads

Encapsulated enzymes were kept at 4 °C for 14 weeks [11]. The beads were weighted and disrupted using trisodium citrate solution (0.2 M, pH 8.2) [9,41] at 4 °C while shaking at 100 rpm for 24 h to release the encapsulated enzyme. The samples were then analyzed for enzyme activity retained over the 14-week storage period as previously described [9].

### 2.8. Statistical Analysis

Statistical analyses were conducted using OriginPro 2019b (9.65) software (Originlab, Northampton, MA, USA). A one-way analysis of variance (ANOVA) was carried out, followed by Tukey’s test to assess differences between the mean values at a 95% confidence level (*p* < 0.05) for all tests.

### 2.9. Ethical Statement

This article does not contain any studies with human participants or animals performed by any of the authors. Rumen liquor (small volumes) was collected as part of the routine collection by the designated technical officer to conduct in vitro dry matter digestibility experiments. All procedures were conducted in accordance with the Australian Code of Practice for the Care and Use of Animals for Scientific Purposes [42]. Approval to collect ruminal fluid from fistulated cows was obtained from the DEECA Agricultural Research and Extension Animal Ethics Committee (approval 2021-13). As no live animals were used in the study, an ethical review was not required. No animal discomfort was caused by sample collection for the study.

## 3. Results and Discussion

### 3.1. Encapsulation Efficiency

**Figure 2:**
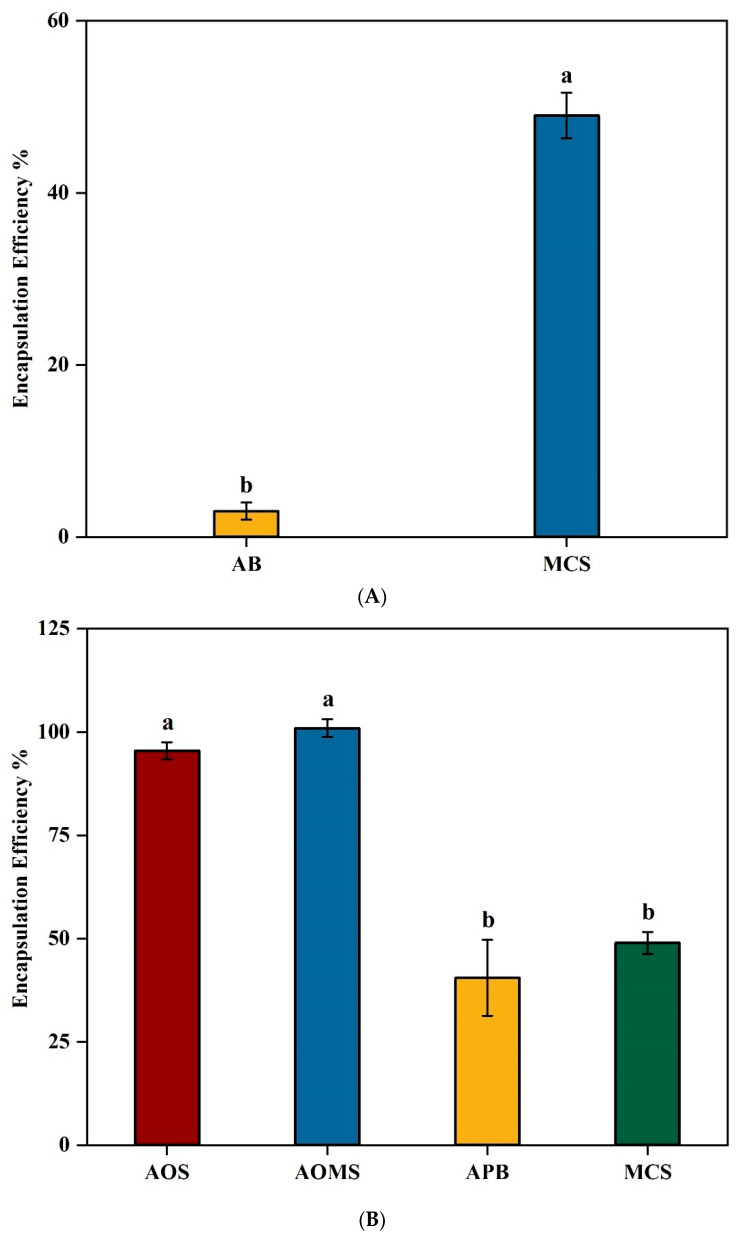
Evaluation of Formulation Strategies to Improve the Encapsulation Efficiency of β-Glucosidase in Alginate Beads. Encapsulation efficiency was calculated based on the activity of the encapsulated β-glucosidase, enzyme activity recovered from the beads, and expressing it as a percentage of the initial β-glucosidase activity in the alginate–enzyme mixture; (**A**) The effect of 0.1% Chitosan addition to 0.1 M CaCl_2_ Solution on Encapsulation Efficiency of β-Glucosidase in Alginate Beads (MCS) compared to alginate–enzyme beads (AB) made with 1% (*w*/*v*) alginate solution (E401) and 0.2 U/mL β-glucosidase in 0.1 M CaCl_2_ alone; (**B**) Impact of Different Additives (Sucrose, Maltodextrin, and Pectin) on the Encapsulation Efficiency of β-Glucosidase in Alginate Beads (MCS). The formulations evaluated include a final concentration of (i) 4% sucrose (AOS), (ii) 4% sucrose and 2% maltodextrin (AOMS), and (iii) 4% pectin (APB), compared to the base formulation (MCS), which contains 1% (*w*/*v*) alginate (E401) and 0.2 U/mL β-glucosidase mixture crosslinked in a solution of 0.1 M CaCl_2_, 0.1% chitosan, and 0.1% Tween20. Different letters indicate statistically significant differences based on one-way ANOVA followed by Tukey’s test (*p* < 0.05).

The alginate microbeads produced in this study, across all formulations, were consistently <1000 µm in size. The encapsulation efficiency (EE) of β-glucosidase in alginate beads was initially low at approximately 3% (Figure 2A) for a 1% (*w*/*v*) alginate (E401) and 0.2 U/mL β-glucosidase mixture, likely due to enzyme leakage during encapsulation. The porous nature of alginate beads can lead to significant enzyme diffusion into the crosslinking calcium chloride solution, as reported in previous studies [11,32,43]. To address this issue, we modified the crosslinking solution by incorporating 0.1% chitosan into 0.1 M CaCl_2_, which significantly improved EE to 49% (*p* < 0.05). This enhancement aligns with findings by Anjani [11], where the addition of 0.1% chitosan increased the EE of flavourzyme from 16% to 72%. Similarly, Thu and Krasaekoopt [9] demonstrated that 0.2% chitosan reduced protease leakage to just 8.1%, emphasizing chitosan’s effectiveness in enzyme retention. The enhanced EE observed in this study can be attributed to chitosan’s ability to interact with alginate as a cationic polymer, binding to the negatively charged alginate chains. Unlike CaCl_2_ alone, chitosan’s larger molecular structure provides superior pore-blocking efficiency, transforming the alginate gel network from macroporous to microporous [11]. Additionally, chitosan crosslinks with alginate via interactions between its amine groups and alginate’s carboxyl groups, reinforcing membrane integrity [9]. Further, low molecular weight chitosan can penetrate the alginate matrix, leading to a denser structure with reduced pore size [9,44]. This structural modification effectively minimizes enzyme leakage, demonstrating that chitosan is a viable strategy for improving β-glucosidase encapsulation efficiency in alginate beads.

Further improvement in the encapsulation efficiency of β-glucosidase was achieved through the incorporation of other carbohydrates (Figure 2B). The control formulation (MCS) included a solution containing 1% (*w*/*v*) high-viscosity alginate (E401) was studied for encapsulating β-glucosidase as described in 2.3 by gelling for 30 min in 0.1 M CaCl_2_ containing 0.1% (*w*/*v*) chitosan and 0.1% (*w*/*v*) tween 20, providing baseline encapsulation efficiency. Three different formulations were evaluated by adding a final concentration of: (i) 4% sucrose (AOS), (ii) 4% sucrose and 2% maltodextrin mixture (AOMS), and (iii) 4% pectin (APB) to the base formulation (MCS). The encapsulation efficiency (EE) data revealed significant differences (*p* < 0.05) among the formulations, as confirmed by one-way ANOVA analysis. These differences can be explained by the distinct properties of the added components. The significant increase (*p* < 0.05) in EE from 49% to 95.5% in AOS can be attributed to its role as a stabilizing and cryoprotective agent. Sucrose helps to preserve the structural integrity of the encapsulated enzyme by reducing the stress associated with drying and bead formation processes. Sucrose stabilizes proteins by preventing denaturation and aggregation during encapsulation, as it forms hydrogen bonds with proteins, protecting their structure in solution [33]. Additionally, sucrose increases the viscosity of the solution, which may improve the bead formation process by creating a more uniform and stable matrix [45]. In a study by Zhang, et al. [46], sucrose, as a filler material, improved structural integrity and encapsulation efficiency within calcium alginate matrices. Moreover, sucrose protects against environmental stresses such as dehydration and temperature fluctuations during encapsulation, aiding in enzyme retention within the beads. Trivedi, et al. [47] demonstrated that adding sucrose at a concentration five times the enzyme’s weight increased residual activity and protein loading in immobilization processes. The stabilizing effect of sucrose likely helped preserve the enzyme’s structure under gas-phase conditions, protecting it from denaturation due to heat or dehydration during the reaction. This suggests that sucrose plays a crucial role in maintaining enzyme stability and bioactivity, especially under conditions involving dehydration, which aligns with its application in our study. The highest EE (100% ± 2.160) was achieved with incorporation of both sucrose and maltodextrin (AOMS). This could be due to maltodextrin’s additional stabilizing effects. Maltodextrin has been shown to significantly increase the encapsulation efficiency from 49% to 100% (*p* < 0.05, *p* = 0.0001) most likely by forming a more robust bead matrix, enhancing gel strength, and reducing bead porosity [9]. Maltodextrin also has cryoprotective properties [33], similar to those of sucrose, which help protect the enzyme’s structure during the encapsulation process by interacting with water molecules during drying or storage. Furthermore, maltodextrin in calcium alginate–maltodextrin beads enhances encapsulation efficiency by increasing the bead size and reducing enzyme leakage, which contributes to better protection of the enzymes from deactivation due to factors like pH and moisture during storage [9]. The combined effects of sucrose and maltodextrin created a synergistic effect, improving both bead integrity and reducing the diffusion of the encapsulated enzyme. In contrast, the addition of pectin (APB) resulted in a reduction in EE from 49% to 40.5% although that was not statistically significant (*p* > 0.05, *p* = 0.1277). This is likely due to pectin’s gelation properties which can interfere with alginate’s crosslinking by calcium ions, resulting in a denser gel structure that restricts enzyme-substrate contact, lowers encapsulation efficiency, and increases enzyme leakage over time [48]. Additionally, the similar negative charges of pectin and alginate may lead to competition for calcium ions, further disrupting effective crosslinking and compromising the stability of the encapsulation [48]. The EE of APB is significantly lower than both AOS and AOMS (*p* < 0.05), indicating that pectin compromises encapsulation efficiency compared to formulations containing sucrose or a combination of sucrose and maltodextrin. However, there was no significant difference between AOS and AOMS (*p* > 0.05, *p* = 0.2293). The higher EE in AOS and AOMS suggests that sucrose and maltodextrin are effective at enhancing the retention of the enzyme within the alginate beads by reinforcing the bead matrix, while pectin destabilizes the structure, leading to lower encapsulation efficiency. The addition of a maltodextrin-sucrose (AOMS) mixture has been selected as the best formulation for enhanced encapsulation efficiency.

### 3.2. In Vitro Rumen Fermentation

**Figure 3:**
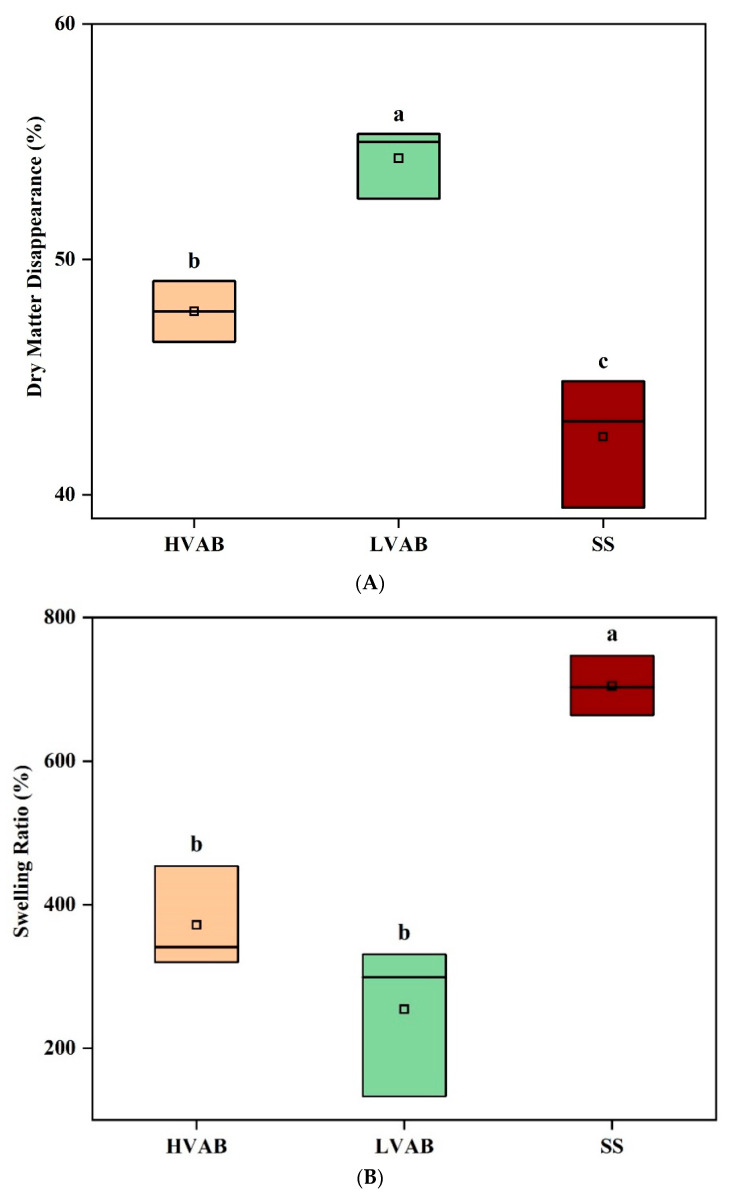

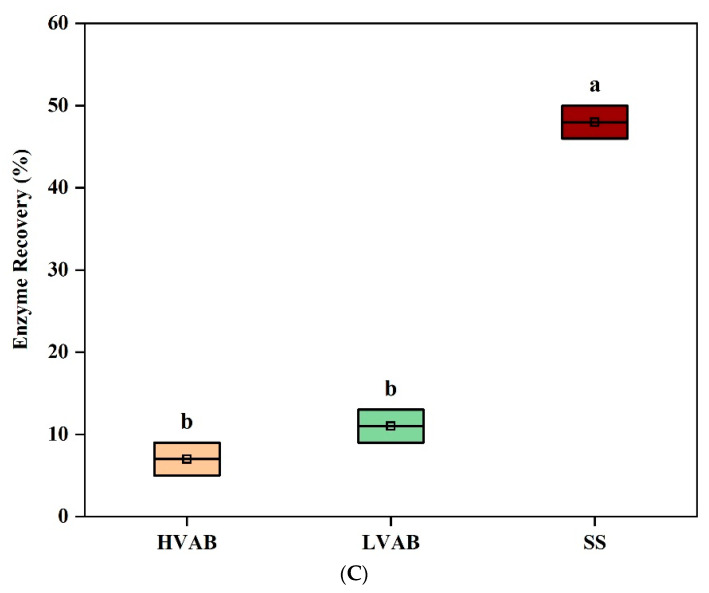
Comparative Analysis of (**A**) dry matter disappearance, (**B**) swelling ratio, and (**C**) enzyme recovery of HVAB (high-viscosity alginate beads incubated in buffered rumen fluid), LVAB (low-viscosity alginate beads incubated in buffered rumen fluid), and SS (low-viscosity alginate beads incubated in buffer only, without rumen fluid). Low-viscosity beads (LVAB) were made from 2% (*w*/*v*) low-viscosity alginate (SF120RB), high-viscosity beads (HVAB) were made from 1% (*w*/*v*) high-viscosity alginate (E401), both containing 2% maltodextrin, 4% sucrose, and 0.2 U/mL enzyme. For crosslinking, a solution containing 0.1 M CaCl_2_, 0.1% chitosan, and 0.1% Tween 20 was used. Different letters indicate statistically significant differences based on one-way ANOVA followed by Tukey’s test (*p* < 0.05).

After finalising the best formulation for enhanced encapsulation efficiency, two types of beads encapsulating the enzyme HVAB (with an EE of 97%) and LVAB (with an EE of 98.5%) and control beads of the same composition with no enzymes, HVABc, and LVABc were produced as explained in Section 2.7.1 and incubated with buffered rumen fluid (39 °C, 50 rpm, 24 h). After incubation, these beads were analysed for dry matter disappearance, enzyme recovery, and swelling ratio. Figure 3A compares the DMD across three different treatments: (i) high-viscosity alginate beads incubated in buffered rumen fluid (HVAB), (ii) low-viscosity alginate beads incubated in buffered rumen fluid (LVAB), (iii) low-viscosity alginate beads incubated in buffer only, without rumen fluid (SS). LVAB showed the highest level of DMD (around 54%, SD = 1.491), followed by HVAB (approximately 48%, SD = 1.289), and SS had the lowest value (around 42%, SD = 2.750). The overall ANOVA analysis reports statistically significant differences between the groups (*p* < 0.05). These results indicate that all formulations were susceptible to some degree of degradation. The Tukey test further clarifies these differences. The comparison between LVAB and HVAB shows the largest mean difference which was statistically significant (*p* < 0.05), and DMD was considerably higher in LVAB compared to HVAB. Similarly, the difference between SS and LVAB was significant (*p* < 0.05). Likewise, the difference in DMD between SS and HVAB was significant (*p* < 0.05), and DMD was higher in both LVAB and HVAB compared to SS. These differences suggest that the presence of rumen fluid significantly influences the extent of DMD. The lower DMD in HVAB compared to LVAB may be attributed to the higher viscosity of the alginate beads. High-viscosity alginate beads likely provide a denser encapsulation matrix, which could reduce the diffusion of rumen microbial enzymes or fermentable substrates through the bead, thus limiting the extent of microbial degradation and fermentation. This would result in less efficient digestion of the encapsulated material. Similar findings were reported by Li, et al. [49], Lee, et al. [50] and Zou, et al. [51], who noted that high-viscosity sodium alginate solutions, due to their larger surface tension, tend to form denser calcium alginate gels with lower swelling capacity. Consequently, those beads retain less water compared to low-viscosity alginate beads, ultimately reducing permeability to external agents. In addition, Afewerki, et al. [52] highlighted how differences in alginate viscosity can influence the behaviour and stability of the resulting gels under physiological conditions. Gels made from low-viscosity alginate are generally less stable and more prone to early breakdown, as they form looser and weaker structures. On the other hand, high-viscosity alginate tends to produce firmer, more durable gels that better withstand environmental stress. However, they may be less permeable as a result, which is desirable from an enzyme protection perspective. The lower DMD in the SS group, where the beads were incubated only in McDougall buffer without rumen fluid, could be due to the absence of microbial and enzymatic action from the rumen fluid contributing to the breakdown of dry matter. Overall, bead viscosity and microbial presence play some role in dry matter disappearance, but substantial mass loss is observed even in the buffer indicating that other factors such as osmotic pressure, pH sensitivity, and polymer water uptake also contribute to material degradation. This observation is consistent with previous studies on pH-sensitive hydrogels [53,54], which have shown that environmental pH and material characteristics can trigger swelling, leading to structural changes even in the absence of enzymatic activity.

The ANOVA results presented for the swelling ratio of HVAB, LVAB, and SS show significant differences between the three groups (*p* < 0.05) (Figure 3B). The means for swelling ratio were 371.67% for HVAB, 254.33% for LVAB, and 704.67% for SS. HVAB, with the higher viscosity alginate beads, exhibited a greater swelling ratio (371.67%) compared to LVAB (254.33%), although the difference was not statistically significant (*p* > 0.05). While previous studies have reported that high-viscosity alginate beads retain less water due to their dense structure [49,50] our findings suggest that in the rumen environment, this denser matrix may help the beads remain intact for longer periods, allowing them to retain more water during incubation. The lower swelling ratio in LVAB could result from the less dense network of low-viscosity alginate beads, making them more prone to early disintegration and water loss during incubation. This was demonstrated by Kong, et al. [55] who studied the properties of alginate-based hydrogels and reported that formulations with a higher content of low molecular weight alginate showed similar elastic behaviour to those made with high molecular weight alginate. However, they were more prone to faster degradation due to a more rapid separation of cross-linked domains over time. The SS group, which is LVAB incubated without rumen fluid, showed the highest swelling ratio (704.67%) (*p* < 0.05). The difference in swelling ratio between the rumen groups (HVAB, LVAB) and the ones without rumen (SS) could be explained by the pH difference after incubation. The final pH in the HVAB and LVAB groups was 6.0 and 5.95, respectively, while in the SS group, it was 6.5 after 24 h incubation. The initial pH of all the samples was 6.8 ± 0.1, but the pH of HVAB and LVAB decreased, likely due to rumen fermentation. Rumen fermentation produces volatile fatty acids, which contribute to the acidification of the environment, thus lowering the pH [56,57]. This pH change is critical because alginate exhibits different swelling behaviours depending on the pH. Alginate gels swell more in higher pH environments due to the ionization of carboxylate groups in the alginate polymer, which increases electrostatic repulsion between polymer chains, thereby enhancing water absorption [58]. This was further supported by Patel, et al. [59] who observed that calcium alginate particles exhibited a noticeably faster swelling rate in simulated intestinal fluid (pH 6.8) than in simulated gastric fluid (pH 1.2), reinforcing the idea that higher pH environments promote ionization within the polymer network, thereby enhancing water uptake. In contrast, at lower pH values, fewer carboxylate groups are ionized, leading to reduced swelling capacity. In the SS group, where the pH remained higher (6.5), the alginate beads could maintain a higher degree of ionization, allowing them to swell more. In contrast, in the HVAB and LVAB groups, the slightly lower pH (6 and 5.95) reduced the ionization of the alginate, limiting their ability to absorb water and swell. This explains the significant difference in swelling ratios between the SS group and the other two groups. These findings are in line with earlier research on pH-sensitive hydrogels, which have shown that changes in pH can significantly affect swelling rate by altering the ionization of functional groups within the polymer network [53,54]. These results highlight the impact of the presence of rumen fluid and buffer on the swelling behaviour of alginate beads.

The ANOVA results for enzyme activity recovery after incubation show significant differences across the three treatments HVAB, LVAB, and SS (Figure 3C). The beads incubated in buffer (SS) had the highest residual enzyme activity with an enzyme recovery (ER) of 48%, followed by LVAB (ER = 11%) and HVAB (ER = 7%). The overall ANOVA analysis indicates statistically significant differences between the groups, with *p* < 0.05. The Tukey test further clarifies these differences. The comparison between SS and HVAB shows the largest mean difference with a (*p* < 0.05), suggesting that enzyme recovery is substantially higher in SS compared to HVAB. Similarly, the difference between SS and LVAB is also significant (*p* < 0.05). However, the difference between LVAB and HVAB was not significant (*p* > 0.05). The SS treatment resulted in substantially higher enzyme recovery compared to beads incubated in the rumen fluid, possibly due to the lack of microbial or enzyme activity, which could degrade the enzyme. In contrast, the lower recovery in both HVAB and LVAB, particularly HVAB, may be due to the interaction between the rumen fluid and the encapsulated enzyme, which may have led to enzyme degradation by the activity of rumen microorganisms and their enzymes [3,60]. The higher viscosity in HVAB may have also restricted enzyme release to the assay matrix, which may have led to the apparently lower recovery compared to LVAB. Alternatively, the increased viscosity may have hindered the diffusion of the substrate into the bead matrix, limiting its access to the encapsulated enzyme. A previous study by Blandino, et al. [61] reported that increasing alginate concentration and the resulting higher viscosity, led to the formation of a more densely cross-linked gel network. This denser structure formed thinner membranes that are less permeable, making it harder for glucose oxidase to diffuse out. Similar findings were observed by Loffredi and and Alamprese [62], who suggested that higher concentrations of sodium alginate slowed down intestinal enzyme diffusion during digestion, likely due to increased viscosity and polysaccharide barriers at the oil/water interface. These findings support the proposition that the increased viscosity of the alginate matrix in HVAB may have formed a physical barrier, thereby limiting the diffusion of the enzyme into the surrounding assay medium. The differences in DMD across the HVAB, LVAB, and SS groups provide important insights into the observed enzyme recovery patterns. The lower enzyme recovery in HVAB and LVAB, particularly in HVAB, can be partially explained by the extent of bead degradation, as indicated by DMD. In HVAB, where DMD is around 48%, and in LVAB, where it is at approximately 54%, significant degradation of the alginate bead matrix likely occurred. This degradation would lead to the release of encapsulated enzyme into the surrounding environment, as stated by Weng, et al. [63]. In the presence of rumen fluid (in both HVAB and LVAB), the enzyme could be exposed to degradation by rumen microorganisms and their enzymes (e.g., proteases) [60,64], and microbial metabolites such as short-chain fatty acids (SCFAs), which can cause a significant reduction in pH during ruminal fermentation [65,66]. This combination of acidic conditions and ruminal microbial activity likely contributes to enzyme degradation [66] and may help explain the reduced recovery observed during in vitro rumen fermentation. In contrast, the SS group, which does not contain rumen fluid, shows DMD (around 42%) and consequently higher enzyme recovery, likely due to the absence of rumen microorganisms and their metabolites. However, partial disintegration of the alginate matrix under buffer conditions may have still contributed to enzyme loss as mentioned earlier, which could explain why enzyme recovery remained relatively low. The high levels of DMD in both HVAB and LVAB point to the need for more robust bead formulations to prevent premature breakdown and enzyme loss. Improved formulations could involve the incorporation of additional crosslinking agents, such as carrageenan and xanthan gum, or the addition of protective layers through polyelectrolyte complexation with chitosan. These modifications could slow down degradation in the rumen environment, preserving both the bead structure and the enzyme encapsulated within. By reducing bead degradation, it would be possible to achieve higher enzyme recovery, as the enzyme would remain protected from the hostile environment of the rumen fluid. In conclusion, the current bead formulations, both HVAB and LVAB, may not be robust enough to prevent degradation and subsequent enzyme loss in the gastrointestinal tract of ruminants. This highlights the necessity for optimizing the bead matrix to protect the enzyme and maintain a more consistent and higher level of enzyme recovery.

### 3.3. Morphological Characterization of Hydrogel Microbeads

**Figure 4:**
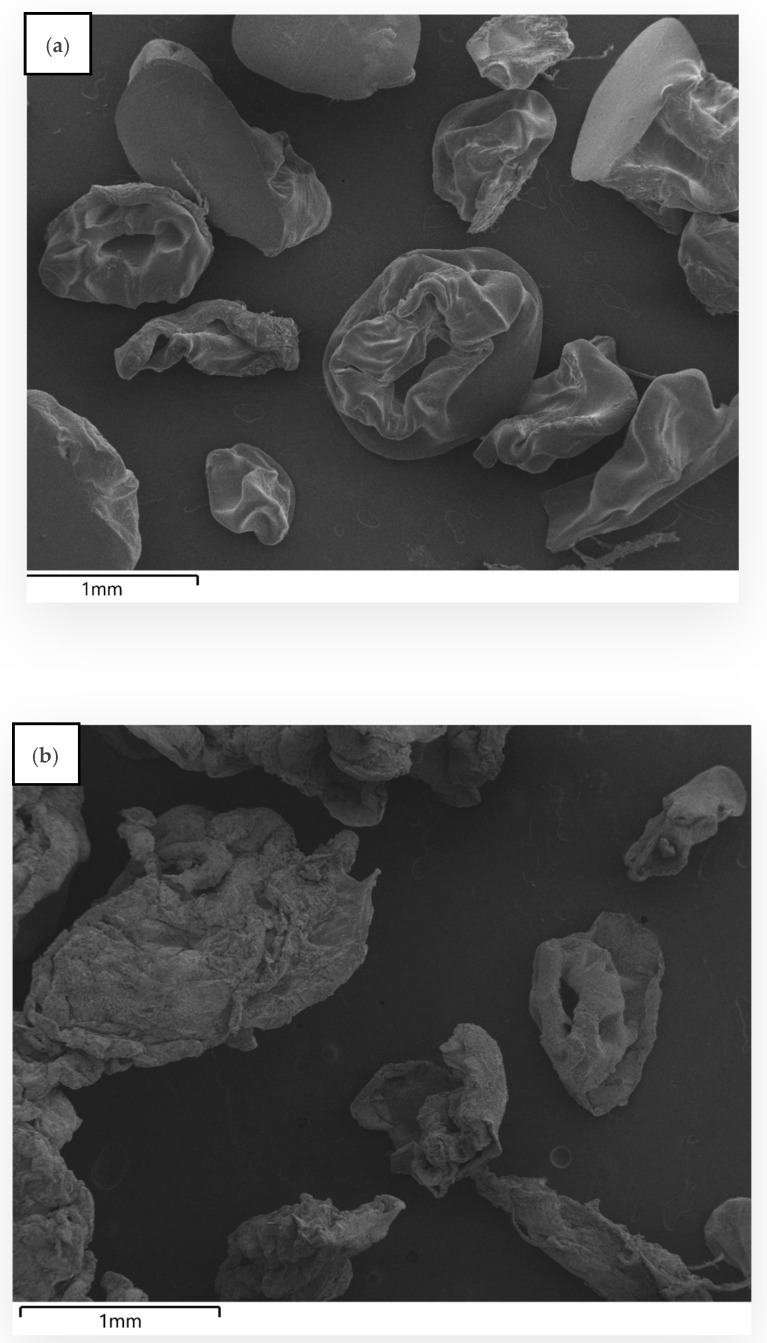

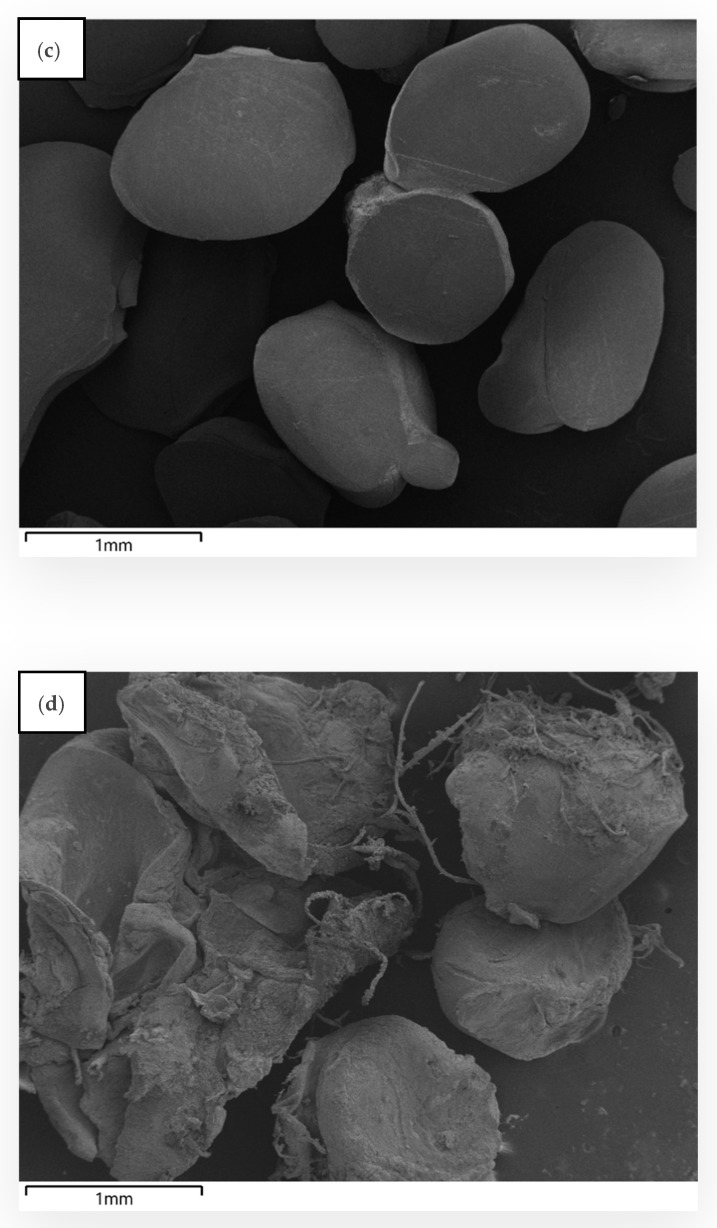

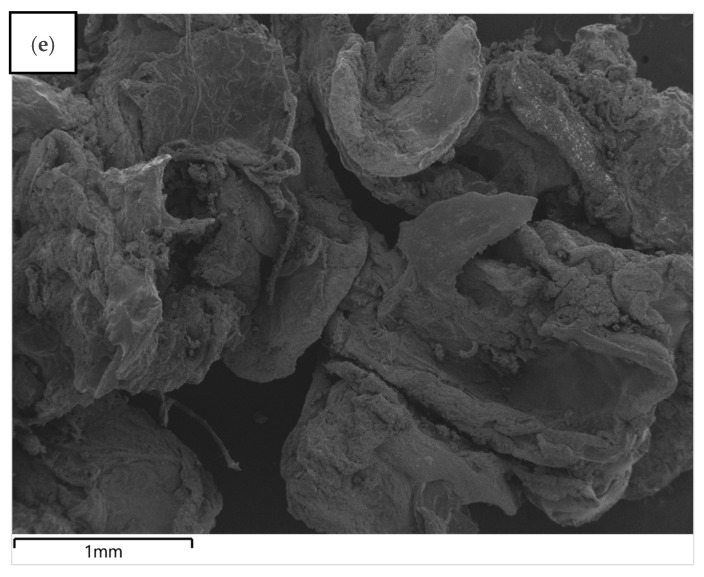
Scanning Electron Microscopy (SEM) images of alginate–chitosan beads. (**a**) HVAB before ruminal incubation; (**b**) HVAB after ruminal incubation; (**c**) LVAB before ruminal incubation; (**d**) LVAB after ruminal incubation; (**e**) LVAB after McDougall buffer incubation. Low-viscosity beads (LVAB) were made from 2% (*w*/*v*) low-viscosity alginate (SF120RB), high-viscosity beads (HVAB) were made from 1% (*w*/*v*) high-viscosity alginate (E401), both containing 2% maltodextrin, 4% sucrose, and 0.2 U/mL enzyme. For crosslinking, a solution containing 0.1 M CaCl_2_, 0.1% chitosan, and 0.1% Tween 20 was used.

In order to determine the structural integrity of the microcapsules before and after in vitro rumen incubation, scanning electron microscopy (SEM) micrograph of the bead samples were taken. Before incubation, the HVAB beads appeared relatively smooth and structured, with wrinkled and slightly deformed shapes (Figure 4a). This is likely due to the drying process, where moisture evaporates from the alginate–sucrose–maltodextrin matrix. During drying, the beads shrink, causing some collapse in structure as water is removed, which results in a wrinkled appearance. Similarly Weng, et al. [67], reported that rapid water evaporation during drying leads to alginate beads shrinkage and surface wrinkling, resulting in dimpled or deformed structures, similar to those observed in HVAB beads. Another study by Hassan, et al. [68] confirmed that sodium alginate hydrogels exhibited wrinkled and porous surfaces due to structural collapse under air-drying conditions. These observations are consistent with findings from a recent study that encapsulated mango peel extract (MPE) into alginate beads. The study reported that freeze-dried MPE-loaded alginate beads exhibited a wrinkled and irregular surface morphology due to moisture loss and matrix shrinkage during the drying process [69]. Post-rumen incubation (Figure 4b), the beads appear disintegrated, showing a much more fragmented and porous structure. The incubation in rumen fluid at pH 6.8 ± 0.1 (39 °C, 24 h) would have exposed the beads to enzymatic and microbial activity. Alginate is typically sensitive to microbial degradation [70]. The ruminal environment is rich in enzymes and microorganisms and that may partly explain the degradation of the beads after incubation. Additionally, alginate gels swell more in a higher pH environment thereby enhancing water absorption which weakens the crosslinked structure [71]. Armutcu and Pişkin [72] showed that drug-loaded alginate beads underwent gradual structural degradation when incubated at pH 7.3 at 37 °C. Structural changes such as shrinkage, porosity, and structural collapse were visible after 6 h of incubation and intensified after 8 days of incubation. By day eight, the beads exhibited a weight loss of up to 27.6%, highlighting alginate’s sensitivity to both near-neutral pH and prolonged incubation. As the bead matrix was partially degraded, 83% of the encapsulated enzyme was released/inactivated. The fragmented nature of the beads suggests that the release of the enzyme would have been gradual as the matrix disintegrated.

Similarly, LVAB before ruminal incubation (Figure 4c) were compact, smooth, and well-formed due to the tight crosslinking and dehydration process [67,68]. However, after ruminal incubation (Figure 4d), the beads appear highly irregular. This external structure likely indicates significant disruption of the outer surface due to the combined effects of microbial degradation, enzymatic activity, and pH-driven swelling [72]. This is in line with the visual observations reported by Gawad and Fellner [73], who noted that during in vitro fermentation with mixed rumen microbes, some beads remained in suspension while others settled to the bottom after 24 h of incubation. Such sedimentation likely reflects structural weakening, swelling, or microbial degradation, which increases bead density and causes them to behave similarly to degraded particulate matter. In contrast, the SS beads (Figure 4e) were swollen which could be attributed to the near-neutral pH of McDougall’s buffer. Zhou, et al. [74] stated that at higher pH levels (e.g., pH 7), alginate beads tend to lose structural stability due to the deprotonation of carboxyl groups within the alginate matrix. This change weakens the polymer network by creating repulsive forces between chains. This promotes increased water uptake, leading to progressive swelling and structural fragmentation. In the study by Zhou, Hu, Wang, Xue and Luo [74], extended incubation over 240 h led to complete disintegration of the beads, highlighting the impact of pH and time on alginate’s physical stability. The absence of microbial and enzymatic degradation allows for better retention of the enzyme within the bead matrix, resulting in higher recovery (48%). However, there was a substantial loss of enzyme activity even in the buffer (62%). This suggests that additional coating or further formulation adjustments are necessary to protect the beads from both pH-induced and microbial-induced breakdown in the rumen.

### 3.4. Stability of Encapsulated Enzymes

High-viscosity alginate beads (HVAB) and low-viscosity alginate beads (LVAB) were stored at 4 °C after air-drying and the shelf-stability of the stored capsules was studied for 14 weeks by measuring the enzyme activity at the end of the storage period. The comparison of enzyme activity in high-viscosity alginate beads (HVAB) and low-viscosity alginate beads (LVAB) over an initial period and after 14 weeks of storage revealed notable differences in their performance (Table 3). Initially, both bead types demonstrated similar enzyme activity, with HVAB showing 0.189 ± 0.036 U/mL and LVAB 0.192 ± 0.029 U/mL, indicating that the encapsulation process was equally effective for both formulations at preserving enzyme functionality. After 3.5 months of storage, enzyme activity was slightly higher (*p* > 0.05) in LVAB (0.214 ± 0.072 U/mL) compared to HVAB (0.199 ± 0.061 U/mL). The slight increase in activity (*p* > 0.05) in both bead types may result from enzyme stabilization within the matrix, where the storage conditions allowed the immobilized enzyme to adopt a more active conformation. This is supported by studies that showed that immobilizing enzymes on solid supports (e.g., beads) can help preserve their active conformation by providing a microenvironment that enhances structural stability during storage [75,76,77]. A recent study by Pradeep Kumar and Sridhar [19] reported that storage of immobilized laccase into agarose beads over 75 days resulted in retaining 63.02% of activity, suggesting that immobilization can offer a stabilizing environment that preserves enzyme functionality over time. Additionally, gradual moisture absorption could enhance enzyme solubility and reactivity [78,79,80]. These findings suggest that both high- and low-viscosity alginate beads were effective in maintaining enzyme stability over the storage period.

## 4. Conclusions

This study demonstrated that modifying alginate-based microencapsulation with chitosan, sucrose, and maltodextrin significantly improved β-glucosidase encapsulation efficiency, achieving up to 100 ± 2.16%. However, in vitro rumen fermentation revealed limitations in enzyme recovery, highlighting the vulnerability of both microcapsules, HVAB and LVAB, to the rumen environment, its microorganisms, their enzymes and metabolites. A comprehensive understanding would have been achieved if in vitro abomasum and intestinal digestion experiments had followed the in vitro rumen fermentation. However, in the present study, our primary focus was to develop a robust microencapsulation system capable of withstanding the rumen environment. Once the formulation of the beads is optimised, we aim to conduct a more comprehensive study to evaluate the stability of the beads during rumen fermentation as well as in vitro abomasum and intestinal digestion. The findings of this study underscore the need for further improvements in bead formulation to address degradation issues and enhance enzyme stability and recovery during passage through the rumen. Improved formulations could involve the incorporation of additional crosslinking agents, such as carrageenan and xanthan gum, or additional protective layers through polyelectrolyte complexation with chitosan. Future work will focus on developing more robust bead matrices by integrating advanced crosslinking techniques or incorporating additional protective layers to better withstand the challenges posed by rumen fermentation and fluctuating pH levels during post-rumen digestion. It is important to acknowledge that in vitro rumen fermentation and digestion studies cannot fully represent the complexity of ruminant gastrointestinal digestion. This limitation will also be considered when designing future validation studies.

## Figures and Tables

**Table 1:** Formulation strategies for enhancing the encapsulation efficiency of β-Glucosidase (0.2 U/mL) using sodium alginate-based matrices.

Formulation	Encapsulant Materials	Gelling Solution
1	AB: 1% (*w*/*v*) sodium alginate solution (E401)	0.1 M CaCl_2_
2	MCS: 1% (*w*/*v*) sodium alginate solution (E401)	0.1 M CaCl_2_ + 0.1% (*w*/*v*) chitosan + 0.1% (*w*/*v*) Tween 20
3	AOS: 1% (*w*/*v*) sodium alginate solution (E401) + 4% (*w*/*v*) sucrose	0.1 M CaCl_2_ + 0.1% (*w*/*v*) chitosan + 0.1% (*w*/*v*) Tween 20
4	AOMS: 1% (*w*/*v*) sodium alginate solution (E401) + 2% (*w*/*v*) maltodextrin (DE-18) + 4% (*w*/*v*) sucrose	0.1 M CaCl_2_ + 0.1% (*w*/*v*) chitosan + 0.1% (*w*/*v*) Tween 20
5	APB: 1% (*w*/*v*) sodium alginate solution (E401) + 4% (*w*/*v*) pectin	0.1 M CaCl_2_ + 0.1% (*w*/*v*) chitosan + 0.1% (*w*/*v*) Tween 20

**Table 2:** Composition of High- and Low-Viscosity Alginate Beads and their Crosslinking Solutions.

Formulation	Encapsulant Materials	Gelling Solution
HVAB	A 1% (*w*/*v*) solution of high-viscosity alginate (E401) + 2% (*w*/*v*) maltodextrin (DE-18) + 4% (*w*/*v*) sucrose + 0.2 U/mL β-glucosidase	0.1 M CaCl_2_ + 0.1% (*w*/*v*) chitosan + 0.1% (*w*/*v*) Tween 20
LVAB	A 2% (*w*/*v*) solution of low-viscosity alginate (SF120RB) + 2% (*w*/*v*) maltodextrin (DE-18) + 4% (*w*/*v*) sucrose + 0.2 U/mL β-glucosidase	0.1 M CaCl_2_ + 0.1% (*w*/*v*) chitosan + 0.1% (*w*/*v*) Tween 20

**Table 3:** Effect of storage for 14 weeks at 4 °C on the activity of β-glucosidase encapsulated in high-viscosity (HVAB) and low-viscosity (LVAB) alginate beads (mean ± SD, n = 3). Values with the same letter in each row are not significantly different (*p* > 0.05).

Beads	Enzyme Activity × 0 (U/mL)	Enzyme Activity × 3.5 Months (U/mL)
High-viscosity alginate beads (HVAB)	0.189 ± 0.036 a	0.199 ± 0.061 a
Low-viscosity alginate beads (LVAB)	0.192 ± 0.029 a	0.214 ± 0.072 a

## Data Availability

The data presented in this study are available on request from the corresponding authors.

## Supplementary Materials

The following supporting information can be downloaded at: https://www.mdpi.com/article/10.3390/bioengineering12121341/s1, Figure S1: PNP standard curve.

## References

[1] Carrillo-Díaz M.I., Miranda-Romero L.A., Chávez-Aguilar G., Zepeda-Batista J.L., González-Reyes M., García-Casillas A.C., Tirado-González D.N., Tirado-Estrada G. (2022). Improvement of ruminal neutral detergent fiber degradability by obtaining and using exogenous fibrolytic enzymes from white-rot fungi. Animals.

[2] Elwakeel E.A., Titgemeyer E.C., Johnson B.J., Armendariz C.K., Shirley J.E. (2007). Fibrolytic Enzymes to Increase the Nutritive Value of Dairy Feedstuffs1. J. Dairy Sci..

[3] Almassri N., Trujillo F.J., Terefe N.S. (2024). Microencapsulation technology for delivery of enzymes in ruminant feed. Front. Vet. Sci..

[4] Gharechahi J., Vahidi M.F., Sharifi G., Ariaeenejad S., Ding X.-Z., Han J.-L., Salekdeh G.H. (2023). Lignocellulose degradation by rumen bacterial communities: New insights from metagenome analyses. Environ. Res..

[5] Bartosz T., Irene T. (2016). Polyphenols encapsulation—Application of innovation technologies to improve stability of natural products. Phys. Sci. Rev..

[6] Morales E., Rubilar M., Burgos-Díaz C., Acevedo F., Penning M., Shene C. (2017). Alginate/Shellac beads developed by external gelation as a highly efficient model system for oil encapsulation with intestinal delivery. Food Hydrocoll..

[7] Ben Messaoud G., Sánchez-González L., Probst L., Jeandel C., Arab-Tehrany E., Desobry S. (2016). Physico-chemical properties of alginate/shellac aqueous-core capsules: Influence of membrane architecture on riboflavin release. Carbohydr. Polym..

[8] Quiroga E., Illanes C., Ochoa N., Barberis S. (2011). Performance improvement of araujiain, a cystein phytoprotease, by immobilization within calcium alginate beads. Process Biochem..

[9] Thu T.T.M., Krasaekoopt W. (2016). Encapsulation of protease from Aspergillus oryzae and lipase from *Thermomyces lanuginoseus* using alginate and different copolymer types. Agric. Nat. Resour..

[10] Siang S., Wai L., Nyam K., Pui L.P. (2019). Effect of added prebiotic (Isomalto-oligosaccharide) and Coating of Beads on the Survival of Microencapsulated Lactobacillus rhamnosus GG. Food Sci. Technol..

[11] Anjani K. (2007). Microencapsulation of Flavour-Enhancing Enzymes for Acceleration of Cheddar Cheese Ripening.

[12] Segale L., Giovannelli L., Mannina P., Pattarino F. (2016). Calcium alginate and calcium alginate-chitosan beads containing celecoxib solubilized in a self-emulsifying phase. Scientifica.

[13] Carvalho Neto J.P.d., Bezerra L.R., da Silva A.L., de Moura J.F.P., Pereira Filho J.M., da Silva Filho E.C., Guedes A.F., Araújo M.J., Edvan R.L., Oliveira R.L. (2019). Methionine microencapsulated with a carnauba (*Copernicia prunifera*) wax matrix for protection from degradation in the rumen. Livest. Sci..

[14] Kim T.-B., Lee J.-S., Cho S.-Y., Lee H.-G. (2020). In Vitro and In Vivo Studies of Rumen-Protected Microencapsulated Supplement Comprising Linseed Oil, Vitamin E, Rosemary Extract, and Hydrogenated Palm Oil on Rumen Fermentation, Physiological Profile, Milk Yield, and Milk Composition in Dairy Cows. Animals.

[15] Yoshimaru T., Takahashi H., Matsumoto K. (2000). Microencapsulation of L-lysine for improving the balance of amino acids in ruminants. J.-Fac. Agric. Kyushu Univ..

[16] Sun Y., Wei Z., Xue C. (2023). Development of zein-based nutraceutical delivery systems: A systematic overview based on recent researches. Food Hydrocoll..

[17] Wei Y., Hu L., Yao J., Shao Z., Chen X. (2019). Facile Dissolution of Zein Using a Common Solvent Dimethyl Sulfoxide. Langmuir.

[18] De Jesús J.A.C., Elghandour M.M.M.Y., Adegbeye M.J., Aguirre D.L., Roque-Jimenez J.A., Lackner M., Salem A.Z.M. (2024). Nano-encapsulation of essential amino acids: Ruminal methane, carbon monoxide, hydrogen sulfide and fermentation. AMB Express.

[19] Pradeep Kumar V., Sridhar M. (2024). Sustainable pretreatment method of lignocellulosic depolymerization for enhanced ruminant productivity using laccase protein immobilized agarose beads. Sci. Rep..

[20] Koyama K.-I., Onishi H., Sakata O., Machida Y. (2009). Preparation and in Vitro Evaluation of Chitosan-coated Alginate/Calcium Complex Microparticles Loaded with Fluorescein-labeled Lactoferrin. Yakugaku Zasshi J. Pharm. Soc. Jpn..

[21] Corrales Ureña Y.R., Lisboa-Filho P.N., Szardenings M., Gätjen L., Noeske P.-L.M., Rischka K. (2016). Formation and composition of adsorbates on hydrophobic carbon surfaces from aqueous laccase-maltodextrin mixture suspension. Appl. Surf. Sci..

[22] Mensink M.A., Frijlink H.W., van der Voort Maarschalk K., Hinrichs W.L.J. (2017). How sugars protect proteins in the solid state and during drying (review): Mechanisms of stabilization in relation to stress conditions. Eur. J. Pharm. Biopharm..

[23] Soltanizadeh N., Mirmoghtadaie L., Nejati F., Najafabadi L.I., Heshmati M.K., Jafari M. (2014). Solid-State Protein–Carbohydrate Interactions and Their Application in the Food Industry. Compr. Rev. Food Sci. Food Saf..

[24] Fallahasghari E.Z. (2024). Electrohydrodynamic Encapsulation to Enhance the Oxidative Stability of Lipophilic Bioactive Compounds. Ph.D. Thesis.

[25] Laureanti E., Paiva T., Jorge L., Jorge R. (2023). Microencapsulation of bioactive compound extracts using maltodextrin and gum arabic by spray and freeze-drying techniques. Int. J. Biol. Macromol..

[26] Phan A.D.T., Adiamo O., Akter S., Netzel M.E., Cozzolino D., Sultanbawa Y. (2021). Effects of drying methods and maltodextrin on vitamin C and quality of Terminalia ferdinandiana fruit powder, an emerging Australian functional food ingredient. J. Sci. Food Agric..

[27] Lee J., Taip F., Abdullah Z. (2018). Effectiveness of additives in spray drying performance: A review. Food Res..

[28] Ćorković I., Pichler A., Ivić I., Šimunović J., Kopjar M. (2021). Microencapsulation of Chokeberry Polyphenols and Volatiles: Application of Alginate and Pectin as Wall Materials. Gels.

[29] Rosmalia A., Permana I., Despal D., Toharmat T., Pambudi F., Arif S. (2023). Effect of Dietary Non-Fiber Carbohydrate Sources and Sulfur Supplementation on in vitro Ruminal Fermentation and Digestibility of the Dairy Ration. Iran. J. Appl. Anim. Sci..

[30] Terefe N.S., Sheean P., Fernando S., Versteeg C. (2013). The stability of almond β-glucosidase during combined high pressure-thermal processing: A kinetic study. Appl. Microbiol. Biotechnol..

[31] Partovinia A., Vatankhah E. (2019). Experimental investigation into size and sphericity of alginate micro-beads produced by electrospraying technique: Operational condition optimization. Carbohydr. Polym..

[32] Kailasapathy K., Perera C., Phillips M. (2006). Evaluation of Alginate-Starch Polymers for Preparation of Enzyme Microcapsules. Int. J. Food Eng..

[33] Farhadian S., Shareghi B., Momeni L., Abou-Zied O.K., Sirotkin V.A., Tachiya M., Saboury A.A. (2018). Insights into the molecular interaction between sucrose and α-chymotrypsin. Int. J. Biol. Macromol..

[34] Tunkala B.Z., DiGiacomo K., Alvarez Hess P.S., Dunshea F.R., Leury B.J. (2022). Rumen fluid preservation for in vitro gas production systems. Anim. Feed Sci. Technol..

[35] Tunkala B.Z., DiGiacomo K., Alvarez Hess P.S., Dunshea F.R., Leury B.J. (2023). In vitro protein fractionation methods for ruminant feeds. Animal.

[36] Tunkala B.Z., DiGiacomo K., Alvarez Hess P.S., Dunshea F.R., Leury B.J. (2023). Impact of rumen fluid storage on in vitro feed fermentation characteristics. Fermentation.

[37] Tilley J., Terry R.A. (1963). A two-stage technique for the in vitro digestion of forage crops. Grass Forage Sci..

[38] Kim I.Y., Ahn G.C., Kwak H.J., Lee Y.K., Oh Y.K., Lee S.S., Kim J.H., Park K.K. (2015). Characteristics of Wet and Dried Distillers Grains on In vitro Ruminal Fermentation and Effects of Dietary Wet Distillers Grains on Performance of Hanwoo Steers. Asian-Australas. J. Anim. Sci..

[39] Shaibani M., Mirshekarloo M.S., Singh R., Easton C.D., Cooray M.C.D., Eshraghi N., Abendroth T., Dörfler S., Althues H., Kaskel S. (2020). Expansion-tolerant architectures for stable cycling of ultrahigh-loading sulfur cathodes in lithium-sulfur batteries. Sci. Adv..

[40] Gunawardana C.A., Kong A., Blackwood D.O., Travis Powell C., Krzyzaniak J.F., Thomas M.C., Calvin Sun C. (2023). Magnesium stearate surface coverage on tablets and drug crystals: Insights from SEM-EDS elemental mapping. Int. J. Pharm..

[41] Azarnia S., Lee B.H., Robert N., Champagne C.P. (2008). Microencapsulation of a recombinant aminopeptidase (PepN) from Lactobacillus rhamnosus S_93_ in chitosan-coated alginate beads. J. Microencapsul..

[42] National Health and Medical Research Council (NHMRC) Australian Code for the Care and Use of Animals for Scientific Purposes. NHMRC 2013. https://www.nhmrc.gov.au/about-us/publications/australian-code-care-and-use-animals-scientific-purposes.

[43] Nguyen L.T., Lau Y.S., Yang K.-L. (2016). Entrapment of cross-linked cellulase colloids in alginate beads for hydrolysis of cellulose. Colloids Surf. B Biointerfaces.

[44] Krasaekoopt W., Bhandari B., Deeth H. (2003). Evaluation of encapsulation techniques of probiotics for yoghurt. Int. Dairy J..

[45] Stojanovic R., Belscak-Cvitanovic A., Manojlovic V., Komes D., Nedovic V., Bugarski B. (2012). Encapsulation of thyme (*Thymus serpyllum* L.) aqueous extract in calcium alginate beads. J. Sci. Food Agric..

[46] Zhang X., Zhao Y., Wu X., Liu J., Zhang Y., Shi Q., Fang Z. (2021). Ultrasonic-assisted extraction, calcium alginate encapsulation and storage stability of mulberry pomace phenolics. J. Food Meas. Charact..

[47] Trivedi A.H., Spiess A.C., Daussmann T., Büchs J. (2006). Effect of additives on gas-phase catalysis with immobilised Thermoanaerobacter species alcohol dehydrogenase (ADH T). Appl. Microbiol. Biotechnol..

[48] Costa J.B., Nascimento L.G.L., Martins E., De Carvalho A.F. (2024). Immobilization of the β-galactosidase enzyme by encapsulation in polymeric matrices for application in the dairy industry. J. Dairy Sci..

[49] Li Y., Kong M., Feng C., Liu W.F., Liu Y., Cheng X.J., Chen X.G. (2012). Preparation and property of layer-by-layer alginate hydrogel beads based on multi-phase emulsion technique. J. Sol-Gel Sci. Technol..

[50] Lee H.Y., Wah C.L., Dolzhenko A.V., Heng P.W.S. (2006). Influence of viscosity and uronic acid composition of alginates on the properties of alginate films and microspheres produced by emulsification. J. Microencapsul..

[51] Zou J., Zhang K., Li W., Qin Y., Sun Q., Ji N., Xie F. (2024). Exploring the role of the thick and dense calcium alginate shell on the anti-digestibility mechanism of corn starch/carboxymethyl cellulose/calcium alginate liquid-core beads prepared by reverse spherification. Food Hydrocoll..

[52] Afewerki S., Sheikhi A., Kannan S., Ahadian S., Khademhosseini A. (2019). Gelatin-polysaccharide composite scaffolds for 3D cell culture and tissue engineering: Towards natural therapeutics. Bioeng. Transl. Med..

[53] Rizwan M., Yahya R., Hassan A., Yar M., Azzahari A.D., Selvanathan V., Sonsudin F., Abouloula C.N. (2017). pH Sensitive Hydrogels in Drug Delivery: Brief History, Properties, Swelling, and Release Mechanism, Material Selection and Applications. Polymers.

[54] Abd El-Ghaffar M.A., Hashem M.S., El-Awady M.K., Rabie A.M. (2012). pH-sensitive sodium alginate hydrogels for riboflavin controlled release. Carbohydr. Polym..

[55] Kong H.J., Kaigler D., Kim K., Mooney D.J. (2004). Controlling Rigidity and Degradation of Alginate Hydrogels via Molecular Weight Distribution. Biomacromolecules.

[56] Owens F.N., Basalan M. (2016). Ruminal fermentation. Rumenology.

[57] Dijkstra J. (1994). Production and absorption of volatile fatty acids in the rumen. Livest. Prod. Sci..

[58] Chen S.-C., Wu Y.-C., Mi F.-L., Lin Y.-H., Yu L.-C., Sung H.-W. (2004). A novel pH-sensitive hydrogel composed of N,O-carboxymethyl chitosan and alginate cross-linked by genipin for protein drug delivery. J. Control. Release.

[59] Patel M.A., AbouGhaly M.H.H., Schryer-Praga J.V., Chadwick K. (2017). The effect of ionotropic gelation residence time on alginate cross-linking and properties. Carbohydr. Polym..

[60] Liang J., Zhang R., Chang J., Chen L., Nabi M., Zhang H., Zhang G., Zhang P. (2024). Rumen microbes, enzymes, metabolisms, and application in lignocellulosic waste conversion—A comprehensive review. Biotechnol. Adv..

[61] Blandino A., Macías M., Cantero D. (2000). Glucose oxidase release from calcium alginate gel capsules. Enzym. Microb. Technol..

[62] Loffredi E., Alamprese C. (2024). Digestion Fate and Food Applications of Emulsions as Delivery Systems for Bioactive Compounds: Challenges and Perspectives. Food Rev. Int..

[63] Weng Y., Yang G., Li Y., Xu L., Chen X., Song H., Zhao C.-X. (2023). Alginate-based materials for enzyme encapsulation. Adv. Colloid Interface Sci..

[64] Brock F.M., Forsberg C.W., Buchanan-Smith J.G. (1982). Proteolytic activity of rumen microorganisms and effects of proteinase inhibitors. Appl. Environ. Microbiol..

[65] Nishihara K., van Niekerk J., Innes D., He Z., Cánovas A., Guan L.L., Steele M. (2023). Transcriptome profiling revealed that key rumen epithelium functions change in relation to short-chain fatty acids and rumen epithelium-attached microbiota during the weaning transition. Genomics.

[66] Ramos S.C., Jeong C.D., Mamuad L.L., Kim S.H., Kang S.H., Kim E.T., Cho Y.I., Lee S.S., Lee S.S. (2021). Diet Transition from High-Forage to High-Concentrate Alters Rumen Bacterial Community Composition, Epithelial Transcriptomes and Ruminal Fermentation Parameters in Dairy Cows. Animals.

[67] Weng Y., Ranaweera S., Zou D., Cameron A.P., Chen X., Song H., Zhao C.-X. (2023). Improved enzyme thermal stability, loading and bioavailability using alginate encapsulation. Food Hydrocoll..

[68] Hassan M., Liu Y., Naidu R., Du J., Qi F., Donne S.W., Islam M.M. (2021). Mesoporous Biopolymer Architecture Enhanced the Adsorption and Selectivity of Aqueous Heavy-Metal Ions. ACS Omega.

[69] Kučuk N., Primožič M., Knez Ž., Leitgeb M. (2024). Alginate Beads with Encapsulated Bioactive Substances from Mangifera indica Peels as Promising Peroral Delivery Systems. Foods.

[70] Tang J.C., Taniguchi H., Chu H., Zhou Q., Nagata S. (2009). Isolation and characterization of alginate-degrading bacteria for disposal of seaweed wastes. Lett. Appl. Microbiol..

[71] Chuang J.-J., Huang Y.-Y., Lo S.-H., Hsu T.-F., Huang W.-Y., Huang S.-L., Lin Y.-S. (2017). Effects of pH on the Shape of Alginate Particles and Its Release Behavior. Int. J. Polym. Sci..

[72] Armutcu C., Pişkin S. (2021). Evaluation of controlled hydroxychloroquine releasing performance from calcium-alginate beads. Hittite J. Sci. Eng..

[73] Gawad R., Fellner V. (2019). Evaluation of glycerol encapsulated with alginate and alginate-chitosan polymers in gut environment and its resistance to rumen microbial degradation. Asian-Australas. J. Anim. Sci..

[74] Zhou M., Hu Q., Wang T., Xue J., Luo Y. (2018). Alginate hydrogel beads as a carrier of low density lipoprotein/pectin nanogels for potential oral delivery applications. Int. J. Biol. Macromol..

[75] Clemons T.D., Evans C.W., Zdyrko B., Luzinov I., Fitzgerald M., Dunlop S.A., Harvey A.R., Iyer K.S., Stubbs K.A. (2011). Multifunctional nanoadditives for the thermodynamic and kinetic stabilization of enzymes. Nanoscale.

[76] Datta S., Christena R., Rajaram Y. (2012). Enzyme immobilization: An overview on techniques and support materials. Biotech.

[77] Chatzikonstantinou A.V., Gkantzou E., Gournis D., Patila M., Stamatis H., Kumar C.V. (2018). Chapter Three—Stabilization of Laccase Through Immobilization on Functionalized GO-Derivatives. Methods in Enzymology.

[78] Ramsey E.D., Guo W., Liu J.Y., Wu X.H., Moo-Young M. (2011). 2.74—Supercritical Fluids. Comprehensive Biotechnology.

[79] Zaks A., Klibanov A.M. (1988). The effect of water on enzyme action in organic media. J. Biol. Chem..

[80] Almeida R.L.J., Santos N.C., Morais J.R.F., de Almeida Mota M.M., da Silva Eduardo R., Muniz C.E.S., de Assis Cavalcante J., da Costa G.A., de Almeida Silva R., de Oliveira B.F. (2024). Effect of freezing rates on α-amylase enzymatic susceptibility, in vitro digestibility, and technological properties of starch microparticles. Food Chem..

