# Supplementary figures and images for: Microencapsulation of β-Glucosidase in Alginate Beads for Post-Rumen Release in Ruminant Gut
Source: Bioengineering (Basel). 2025 Dec 9;12(12):1341. doi: 10.3390/bioengineering12121341 (PMC12729419; doi:10.3390/bioengineering12121341)

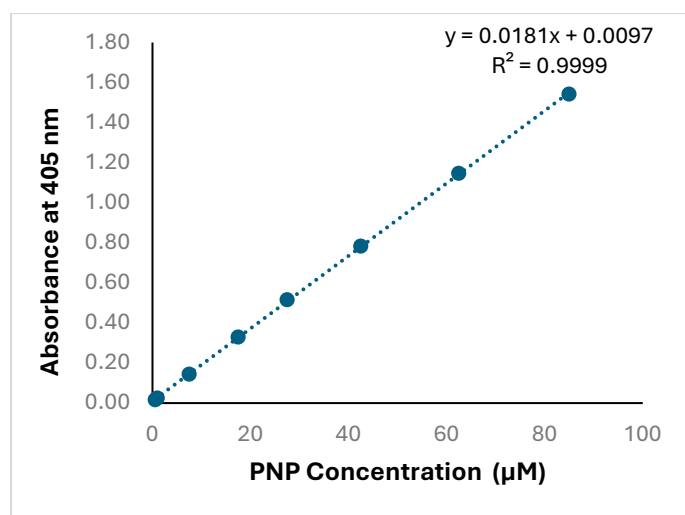

**Figure S1.** PNP standard curve

Supplement: Supplementary file 1 [file bioengineering-12-01341-s001.zip › bioengineering-3971016-supplementary.pdf]
